# A prospective phase-II trial of biweekly docetaxel plus androgen deprivation therapy in patients with previously-untreated metastatic castration-naïve prostate cancer

**DOI:** 10.1186/s12885-021-09018-6

**Published:** 2021-11-29

**Authors:** Seonggyu Byeon, Hongsik Kim, Hwang Gyun Jeon, Seong Il Seo, Seong Soo Jeon, Hyun Moo Lee, Soon Il Lee, Se Hoon Park

**Affiliations:** 1 Department of Internal Medicine, Chungbuk National University Hospital, Chungbuk National University College of Medicine, Cheongju, South Korea; 2 Division of Hematology-Oncology, Department of Medicine, Sungkyunkwan University School of Medicine, Samsung Medical Center, Seoul, South Korea; 3 Department of Urology, Samsung Medical Center, Sungkyunkwan University School of Medicine, Seoul, South Korea; 4 Internal Medicine, Dankook University College of Medicine, Cheonan, Chungcheongnam-do Korea

## Abstract

**Introduction:**

The aim of this prospective phase II study was to evaluate the efficacy and safety of biweekly docetaxel plus androgen-deprivation therapy (ADT) in patients with metastatic castration-naïve prostate cancer (mCNPC).

**Patients and methods:**

Patients with histologically-proven, previously-untreated mCNPC received ADT plus docetaxel, 40 mg/m^2^. Docetaxel was repeated every 2 weeks, up to 12 cycles. Endpoints included castration-resistant prostate cancer (CRPC)-free survival, prostate-specific antigen (PSA) response, and safety.

**Results:**

A total of 42 patients were registered and analyzed for final outcomes. Of the 42 patients, 36 (86%) completed the 12 planned cycles of docetaxel plus ADT. During a median follow up of 25 months, all but two patients (95%) achieved a PSA response with a nadir PSA level of 0.42 ng/ml (range 0.01–1280.87). The median CRPC-free survival was 26.4 months (95% confidence interval [CI] 20.9–32.0) with a one-year CRPC-free rate of 79% (33 patients, 95% CI 66–91). Multivariable analysis revealed that the performance status of the Eastern Cooperative Oncology Group 0 was independently associated with longer CRPC-free survival (hazard ratio [HR] 0.27, 95% CI 0.07–0.99). The most common adverse events of any grade were anemia (95%), followed by nail changes (33%), fatigue (29%), and oral mucositis (26%). Severe (grade 3 or higher) adverse events were infrequent: pneumonitis (*n* = 2), diarrhea (*n* = 1), and neutropenia (*n* = 1).

**Conclusion:**

Our results suggest that biweekly docetaxel plus ADT is feasible, and clinical efficacy does not seem to be compromised compared to a standard triweekly docetaxel 75 mg/m^2^ plus ADT regimen.

## Introduction

Globally,prostate cancer is a leading cause of cancer-related deaths, with more than 10,000 cases diagnosed and 1821 deaths annually in Korea alone [1]. Prostate cancer is an androgen-dependent disease, and the main treatment in the control of prostate cancer growth is androgen-deprivation therapy (ADT), including a luteinizing hormone-releasing hormone (LHRH) agonist/antagonist (medical castration) or bilateral orchiectomy (surgical castration) [2]. Based on findings from recent clinical trials [3–5], current guidelines have established the addition of docetaxel or modern androgen axis targeting agents (abiraterone acetate, and enzalutamide) to ADT as the standard of care for patients with metastatic castration-naïve prostate cancer (mCNPC) [6, 7]. While the evidence is compelling when analyzed by the volume of disease or risk, the long term follow-up of the largest trial confirmed the benefit of adding docetaxel to ADT persisted regardless of disease burden [8].

A major challenge in the management of mCNPC is balancing the toxicity of therapy with clinical benefit. In most trials involving docetaxel, patients received 6 cycles at 75 mg/m^2^ every 3 weeks with ADT. Although the triweekly regimen was active to mCNPC [3, 4], relatively high incidence of febrile neutropenia was regarded as one of the major obstacles. As an alternative treatment, a weekly or biweekly docetaxel administration has been considered as a way of attractive regimen with reduced hematological toxicity.

In a phase III trial comparing docetaxel 50 mg/m^2^ every other week to docetaxel 75 mg/m^2^ every third week in metastatic castration-resistant prostate cancer (mCRPC) [9], the biweekly regimen was associated with reduced hematological toxicities, fatigue being the most frequent adverse event, and a significantly longer time to treatment failure and improved overall survival, which may be related to a greater dose-intensity. These findings, supported by our retrospective studies performed in mCRPC as well as in mCNPC settings, suggest that the tolerability of docetaxel could be further improved [10, 11]. We thus designed the present prospective phase 2 study to evaluate the safety and efficacy of a biweekly schedule of docetaxel added to ADT as first-line treatment in mCNPC.

## Patients and methods

### Patients

In the present single-center, prospective phase II study, key eligibility criteria included histologically confirmed mCNPC. Other inclusion criteria were as follows: age 20 years or older; an Eastern Cooperative Oncology Group (ECOG) performance status of 0 or 1; no prior systemic treatment; at least one measurable metastatic lesion or evaluable bone lesions; and adequate organ function. Eligible patients were selected by a multidisciplinary urologic oncology tumor board composed of urologists, radiologists, pathologists, and medical oncologists. The study protocol (ClinicalTrials.gov, NCT03061643 [23/02/2017]; CRIS.nih.go.kr, KCT0003546) was approved by the institutional review board of Samsung Medical Center (SMC 2017–01–005-005, Seoul, Korea). All patients provided written informed consent. All study procedures were conducted in accordance to good clinical practice, and the Declaration of Helsinki.

### Treatment and evaluation

Following 4 weeks of ADT treatment, patients received docetaxel 40 mg/m^2^ intravenously on day 1 with standard premedication. Docetaxel was repeated every 2 weeks, for up to 12 cycles in the absence of unacceptable toxicity or progressive disease. Supportive care, including the administration of anti-emetics, blood products, bisphosphonates or denosumab, and the use of analgesics, was given if judged appropriate by the investigator. Primary granulocyte colony stimulating factor (G-CSF) prophylaxis was not permitted unless the patient had history of febrile neutropenia.

Patients were seen every 2 weeks; during the visits, a physical examination, blood counts and chemistries, PSA level, and adverse events were assessed. Adverse events were collected and graded according to the National Cancer Institute Common Terminology Criteria v4. Radiologic evaluation of tumor burdens was performed every 8 weeks by computed tomography (CT) scans and bone scintigraphy or using the same tests that were used for initial staging. If a patient had bone-only metastases, radiologic responses were categorized as complete response, stable disease, or progressive disease. After completion of the study treatment, these assessments and administration of ADT were performed according to the institutional guidelines.

### Statistics

The primary endpoint of the study was mCRPC-free survival. The development of mCRPC was defined in accordance with the Prostate Cancer Working Group 2 (PCWG2) criteria [12]. In brief, progressive disease in the setting of medical or surgical castration was defined by one or more of the followings: (1) PSA progression defined by a minimum of 2 rising PSA values from 3 consecutive tests; (2) nodal and/or visceral disease progression as defined by RECIST; (3) bone progression defined by 2 or more new lesions. In addition, if a patient discontinued docetaxel and started a new systemic therapy for any reasons or died from any causes, whichever occurred first, the date was recorded and it was considered an mCRPC event. Secondary endpoints included PSA response, radiologic response, and safety. PSA response was defined as a 50% or more decline in serum PSA from baseline. Those who received at least one dose of study treatment and had follow-up were assessed for safety. A follow-up duration was calculated from the date of the study enrollment until death or the last follow-up. Time for follow-up and survival were calculated using the Kaplan-Meier method. A Cox proportional hazards regression model was used to identify independent factors associated with prolonged mCPRC-free survival. Variables for regression analysis included age, ECOG performance status, disease status, previous therapy, Gleason score, presence of visceral metastasis, or baseline PSA. Visual assessment of Kaplan-Meier method was used to verify the PH assumption. Two-sided *P* values less than 0.05 were considered to indicate significance. All statistical analyses were performed using SPSS v.18 for Windows.

Sample size calculation is based on single-stage phase II designs. Detected between 40 and 60% CRPC-free rates at one-sided significance level of 0.05, 1-β power 0.80. A study requires 42 subjects to decide whether the proportion responding, P, is less than or equal to 0.400 or greater than or equal to 0.600. If the number of responses is 23 or more, the hypothesis that *P* < = 0.400 is rejected with a target error rate of 0.050 and an actual error rate of 0.038. If the number of responses is 22 or less, the hypothesis that *P* > = 0.600 is rejected with a target error rate of 0.200 and an actual error rate of 0.197. Assuming 10% drop-out, it is planned to recruit up to 47 patients in for this study. The final efficacy analysis will be performed according to the intent-to-treat (ITT) population.

## Results

Between Aug. 2018 and Sep. 2019, a total of 42 patients entered the study and received first-line docetaxel plus ADT. Baseline characteristics of patients are summarized in Table 1. The median age at study entry was 68 years (range 55–80). Most (86%) patients had mCNPC at initial diagnosis, while other patients (14%) had local disease at initial diagnosis but experienced disease progression despite local treatment. These patients were enrolled to current study at the time of mCNPC diagnosis. The median Gleason score was 9 (range 7–10). Bone metastases were noted in 34 (81%) patients, and the median number of sites of bone lesions was 2 (range 2–6). Fifteen (36%) patients had visceral metastases, with lung (*n* = 10) and liver (*n* = 5) being the most frequent sites.

**Table 1:** Baseline Characteristics of the Total Population

Baseline Characteristics	*N* = 42 (%)
Age, years, median (range)	**68 (55–80)**
ECOG performance status	
0	**12 (29)**
1	**30 (71)**
Disease status	
Recurrent after local therapy	**6 (14)**
Initially metastatic	**36 (86)**
Previous treatment	
Prostatectomy	**5 (12)**
Prostate radiotherapy	**4 (10)**
Gleason Score	
7	**3 (7)**
8	**14 (33)**
9	**23 (55)**
10	**2 (5)**
Metastasis	
Bone	**34 (81)**
Lymph node	**24 (57)**
Visceral	**15 (36)**
* Bone+Lymph node	**14 (33)**
* Bone+Lymph node+Visceral	**5 (12)**
Number of metastatic sites	
1	**19 (45)**
2	**16 (38)**
3 or more	**7 (17)**
Volume of metastases (according to CHAARTED trial) [3]	
Low	**8 (19)**
High	**34 (81)**
Risk of metastases (according to LATITUDE trial) [5]	
Low	**12 (29)**
High	**30 (71)**
Baseline Laboratory Finding, Median (range)	
Hemoglobin, g/dl	**12.75 (8.5–17.5)**
Alkaline phosphatase, U/L	**1570 (450–3070)**
Absolute neutrophil count, × 10^3^/μL	**3270 (1770–7880)**
Neutrophil lymphocyte ratio	**2.15 (0.8–6.1)**
PSA at screening, ng/ml	**66.90 (0.04–2759.44)**

*ECOG* Eastern Cooperative Oncology Group, *PSA* Prostate specific antigen

*Because patients could have metastases at multiple sites, the total numbers of metastases are larger than the number of patients

### Safety

Of the 42 enrolled patients, 36 (86%) completed the planned 12 cycles of docetaxel plus ADT: 3 patients discontinued because of disease progression and 3 because of adverse events (pneumonitis, *n* = 2; death, *n* = 1). A 73-year-old patient died following a fall in his home shortly after the fifth cycle of docetaxel. Biweekly docetaxel was generally well tolerated (Tables 2). The most commonly observed adverse events of any grade were anemia (95%), nail changes (33%), fatigue (29%), and oral mucositis (26%). Severe (grade 3 or higher) adverse events were infrequent: pneumonitis (*n* = 2), diarrhea (*n* = 1), and neutropenia (*n* = 1). Any case of febrile neutropenia had not been reported until data cutoff date. Among the 465 cycles of biweekly docetaxel for all patients, 18 and 19 doses were delayed and reduced, respectively. As a dose intensity of docetaxel 20 mg/m^2^/week was planned, the relative dose intensity was 98% (95% confidence interval [CI] 86–100).

**Table 2:** Treatment-Related Adverse Events

Adverse events	All Grades	%	Grade ≥ 3	%
Nail change	**14**	**33.3**	**0**	
Fatigue	**12**	**28.6**	**0**	
Mucositis	**11**	**26.2**	**0**	
Alopecia	**9**	**21.4**	**0**	
Neuropathy	**8**	**19.0**	**0**	
Constipation	**7**	**19.0**	**0**	
Pain	**7**	**16.7**	**0**	
Insomnia	**6**	**14.3**	**0**	
Rash	**5**	**11.9**	**0**	
Anorexia	**5**	**11.9**	**0**	
Lacrimation	**5**	**11.9**	**0**	
Dizziness	**3**	**7.1**	**0**	
Diarrhea	**3**	**7.1**	**1**	**2.4**
Localized Edema	**2**	**4.8**	**0**	
Chest discomfort	**2**	**4.8**	**0**	
Pruritus	**2**	**4.8**	**0**	
Dyspnea	**2**	**4.8**	**0**	
Facial flushing	**2**	**4.8**	**0**	
Pneumonitis	**2**	**4.8**	**2**	**4.8**
Nocturia	**1**	**2.4**	**0**	
Myalgia	**1**	**2.4**	**0**	
Sore throat	**1**	**2.4**	**0**	
Anemia	**40**	**95.2**	**0**	
Lymphopenia	**13**	**31**	**2**	**4.8**
Neutropenia	**11**	**26.2**	**1**	**2.4**
Thrombocytopenia	**8**	**19**	**0**	
AST elevation	**1**	**2.4**	**0**	
ALT elevation	**1**	**2.4**	**0**	

*AST* aspartate aminotransferase, *ALT* alanine aminotransferase

### Outcomes

**Fig. 1:**
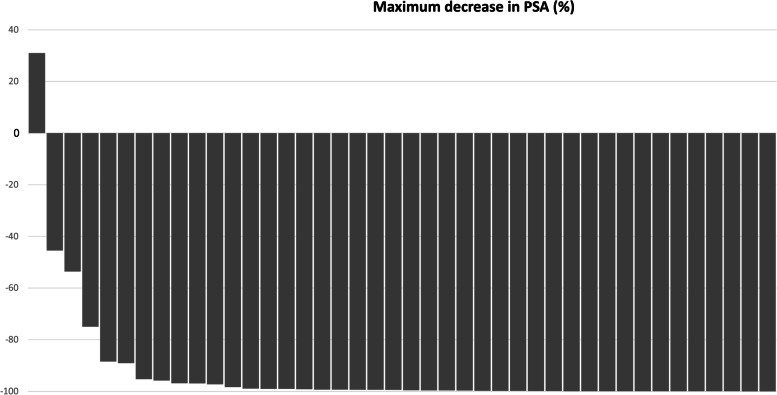
Waterfall plot of nadir PSA in chemotherapy-naïve prostate cancer patients (*N* = 42) treated with biweekly docetaxel plus androgen-deprivation therapy

**Fig. 2:**
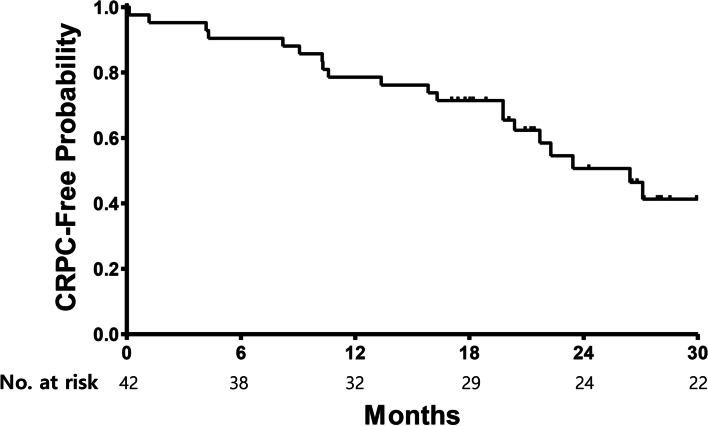
Kaplan-Meier curves for CRPC-free survival of chemotherapy-naïve prostate cancer patients (*N* = 42) treated with biweekly docetaxel plus androgen-deprivation therapy. CRPC: castration resistant prostate cancer

All but two patients (95%) achieved a PSA response (Table 3). After 6 months of biweekly docetaxel, a median PSA nadir of 0.42 ng/ml (range 0.01–1280.87) was achieved. Of note, a decrease in PSA was observed in all but one patient. (Fig. 1) A 65-year-old man with multiple bone and lymph node metastases showed a rapid deterioration of symptoms and PSA progression after 2 cycles. A retrospective review of his tumor biopsy revealed a Gleason score 9 (5 + 4) adenocarcinoma in 12/12 cores with ATM (G494D) somatic mutation. Among the 31 patients who had measurable disease at baseline, 29 had an objective response per RECIST. In an intent-to-treat principle, the response rate was 69% (95% CI 55–83). An additional 11 patients had stable disease, leading to a disease control rate of 95%. With a median follow-up of 25 months, the median mCRPC-free survival was 26.4 months (95% CI 20.9–32.0, Fig. 2). The one-year mCRPC-free rate was 79% (33 patients, 95% CI 66–91).

**Table 3:** Treatment outcomes of biweekly Docetaxel plus ADT

Treatment outcomes	*N* = 42 (%)
PSA response at 12 weeks	
≥ 30% decline	**40 (95.2%)**
≥ 50% decline	**40 (95.2%)**
≥ 90% decline	**28 (66.7%)**
Objective Response Rate	**69%**
mCRPC-free survival, Median (95% CI)	**26.4 months (20.9–32.0)**
Overall Survival, Median (95% CI)	**Not reached**

*mCRPC* metastatic castration resistant prostate cancer, *CI* confidence interval

To explore predictive factors for a prolonged mCRPC-free survival with biweekly docetaxel plus ADT, we performed a multivariable regression analysis using known clinical and laboratory parameters. While mCRPC-free survival was independently associated with an ECOG performance status of 0 (hazard ratio [HR] 0.27, 95% CI 0.07–0.99), it was not influenced by age, disease status, previous therapy, Gleason score, presence of visceral metastasis, or baseline PSA. Further analysis regarding mCRPC development modified by interaction between parameters did not find significant interaction.

## Discussion

The present prospective phase II study of biweekly docetaxel (40 mg/m2) plus ADT for the treatment of mCNPC shows the feasibility of using a more frequent, but lower dosing docetaxel treatment with clinical outcomes consistent with previous phase III trials conducted with the standard docetaxel regimen. PSA (95%) and radiologic (69%) responses were encouraging, and the toxicity profile was mild and easily manageable. The results compared well with our previous retrospective study [11], where the same docetaxel regimen was used in the treatment of high-risk mCNPC.

In the treatment of mCNPC, we already have more than a few treatment options including ADT plus docetaxel, abiraterone acetate, apalutamide, and enzalutamide [6, 7, 13]. Although no direct comparisons have been conducted between the available options for mCNPC, STAMPEDE investigators provided results from a retrospective analysis of 566 patients who had been treated in the docetaxel and the abiraterone arms in the overlapping period from Nov 2011 and Mar 2013 [14]. With a median follow-up of 4 years, they found no difference in prostate cancer-specific survival (HR 1.02, 95% CI 0.70–1.49) between the two groups. Although abiraterone treatment showed better failure-free survival (HR 0.51, 95% CI 0.39–0.67) and progression-free survival (HR 0.65, 95% CI 0.48–0.88), OS was favored docetaxel treatment (HR 1.16, 95% CI 0.82–1.65). This discrepancy might be originated from larger number of non-cancer related deaths in the abiraterone arm than docetaxel arm (19% versus 9%, respectively).

One of the main differences between docetaxel and abiraterone or androgen receptor-targeting agents was the treatment duration. Patients with mCNPC receive ADT plus either < 6 months of docetaxel or long-term (i.e., until progression or unacceptable toxicities) abiraterone. If a patient with mCNPC receives ADT plus abiraterone or enzalutamide, treatment has to be continued for years, during which the patient is constantly exposed to therapy and thus more prone to cumulative toxicity. Conversely, if a patient receives ADT plus docetaxel, acute hematologic toxicities can sometimes be severe [3, 4]. A real-world, retrospective study showed that the incidence of febrile neutropenia was 18%, and docetaxel dose reductions and delays were required in 39 and 16% of cases. The rates were even higher in Asian patients, representing 64–97% of patients treated with docetaxel plus ADT experienced grade > 3 neutropenia [15, 16]. Considering more than half of cases are diagnosed in elderly patients, hematologic toxicities of docetaxel can be a major hurdle for general application to mCNPC. In the present study, we tested docetaxel at a lower (20 mg/m^2^/week) dose, based on a pharmacokinetic study conducted in Japan [17] and the belief that safety and tolerability are indispensable in the treatment of cancer patients in a palliative setting [18]. As expected, our biweekly regimen was well-tolerated and appeared effective in the treatment of mCNPC.

There are several limitations in this study, including being a single-center trial with a small sample size. Additionally, our results are limited to mCNPC patients with high-volume disease. Although the study protocol permitted inclusion of all patients with mCNPC irrespective of risk group, recruitment through a multidisciplinary tumor board led to inclusion of patients with extensive bone and/or visceral metastases. Despite these limitations, the prospective study design and demonstration of comparable clinical efficacy support consideration of the biweekly 40 mg/m^2^ schedule for patients with mCNPC treated with ADT plus docetaxel.

## Conclusion

Docetaxel administered 75 mg/m^2^ every 3 weeks is a current standard regimen in the treatment of mCNPC. However, treatment-related adverse events are common in elderly and fragile patients who receive the standard regimen. Biweekly 40 mg/m^2^ docetaxel plus ADT is feasible, and clinical efficacy does not seem to be compromised. Our results suggest that implementation of this approach in select patients may result in reduced toxicity, improved quality of life, and potentially improved clinical outcomes.

## Data Availability

The datasets of current study are available from the corresponding authors on reasonable request.
